# The Cure of Sciatica and Violent Neuralgia by Hypodermic Injections of Ether

**Published:** 1879-02-20

**Authors:** C. G. Comegs


### *THE CURE OF SCIATICA AND VIOLENT NEURALGIA BY HYPODERMIC INJECTIONS OF ETHER.*

BY C. G. COMEGS, M.D.

In your number of July 6, *ultimo*, I published some cures of sciatica by subcutaneous injections of ether.

In two or three instances I have had occasion to prove its efficacy since that time; and now will briefly state the successful use of ether by a well-known physician of Ottawa, Illinois. He saw the article in the Pharmaceutical News copied from your journal, and wrote to me Nov. 7th, stating that, "I have suffered for two months with acute sciatica in the right hip and leg. I am only able to walk with a crutch, and still suffer much pain especially at night; but I have not contracted the

'opium habit.' I have used all the usual remedies including colchicum, iodide of potassium, quinine, electricity, hypodermic injections of morphia, atropine, cupping, etc. I think my case is like that of the Kentuckian which you detailed. I have been temperate, always healthy and engaged in active practice for twenty-five years in this place—have never been sick, until this terrible calamity overtook me. I came of a rheumatic family, but have never had an acute attack."

In a letter to me dated December 25th, he says:

"Immediately after receiving your letter, I went to my brother, a physician of Aurora, for treatment by ether injections. He used it according to your directions, inserting it back of the great trochanter of the right thigh; using 20 drops the first time, 25 the second, and then 30 drops, or a syringe full night and morning for two days longer, three days in all. It caused great pain for a few moments only, and I distinctly tasted it in my mouth. This was followed by an abatement of the pain; but I received no other immediate relief; I was still hopping and using my cane to lean upon. I used no more injections for fear of ulcers or other local lesion (which did not follow, however), and I waited, growing gradually better every day, until now I feel about as well as ever, do not use my cane, and am visiting patients as usual; taking a little care of myself and not answering night calls. Some hardening of the cellular tissue followed the injections, but they have about disappeared."

I have had recently a violent case of neuralgia of the branch of the circumflex nerve of the right shoulder, as it accompanies the long head of the biceps into the joint. Before I saw the patient he had taken a free dose of morphia, and was so much prostrated by it that he had sent hastily in the night for a physician next door. At first when I saw him I used locally, water as hot as could be borne, which gave much relief; at the same time I gave 15 grs. of quinine night and morning with an ounce of whisky every three hours. Great alleviation followed this treatment, but no cure. Then after three days, I injected 30 drops of ether with the promptest relief. I followed it up twice more, at intervals of twelve hours, with complete success; my patient resuming his business after a confinement of six days, and has had no relapse.

The injections were made over the deltoid muscle on the line of tendon of the biceps. Neuralgia of the circumflex is often intensely severe in those cases of inebriation whether of the alcoholic or opiate character, where we cut off suddenly and completely the stimulant: as great suffering as I have ever witnessed has been in such cases; but an acute case of neuralgia of the circumflex of the shoulder, I have never seen but once, besides the case described above.

I would have no hesitation now in using in such cases, without delay, the hypodermic injections of ether.

From the anatomical relations of the circumflex nerve, we would be justified in believing that a rheumatismal affection of the fibrous tissues about the track of the tendon of the biceps would, by pressure, cause the great suffering of the nerve, but in these cases there are no other manifestations of rheumatism and no fever, so, as I think, it is not necessary to use any constitutional treatment, but simply the local application of ether.

I think it may now be considered as a settled fact that the use of ether hypodermically, is a safe, speedy and certain *relief* for sciatica, tic douloureux, coccydinia, etc.—*Lancet and Clinic.*

